# Inhibition of Lipid Accumulation and Adipokine Levels in Maturing Adipocytes by *Bauhinia rufescens* (Lam.) Stem Bark Extract Loaded Titanium Oxide Nanoparticles

**DOI:** 10.3390/molecules26237238

**Published:** 2021-11-29

**Authors:** Ghedeir M. Alshammari, Abu ElGasim A. Yagoub, Pandurangan Subash-Babu, Amro B. Hassan, Doha M. Al-Nouri, Mohammed A. Mohammed, Mohammed A. Yahya, Rasha Elsayim

**Affiliations:** 1 Department of Food Science and Nutrition, College of Food and Agricultural Sciences, King Saud University, P.O. Box 2460, Riyadh 11451, Saudi Arabia; aghedeir@ksu.edu.sa (G.M.A.); sbpandurangan@ksu.edu.sa (P.S.-B.); ahassan2ks.c@ksu.edu.sa (A.B.H.); dr_nouri@ksu.edu.sa (D.M.A.-N.); 442106434@student.ksu.edu.sa (M.A.M.); 441106332@student.ksu.edu.sa (M.A.Y.); 2 Department of Microbiology, College of Sciences, King Saud University, P.O. Box 2460, Riyadh 11451, Saudi Arabia; 438203748@student.ksu.edu.sa

**Keywords:** *Bauhinia rufescens*, titanium oxide nanoparticles, green synthesis, eco-toxicity, inflammation, obesity

## Abstract

The present study reports a cost-effective, environmentally friendly method to increase the bioavailability and bio-efficacy of *B. rufescens* stem bark extract in the biological system via functional modification as *B. rufescens* stem bark nanoparticles (BR-TO_2_-NPs). The biosynthesis of BR- -NPs was confirmed by UV-visible (UV-vis) and Fourier-transform infrared (FT-IR) spectroscopy, transmission electron microscopy (TEM), and X-ray diffraction analyses. The shifts in FT-IR stretching vibrations of carboxylic and nitro groups (1615 cm^−1^), the O–H of phenolics or carboxylic acids (3405 cm^−1^), alkanes, and alkyne groups (2925 and 2224 cm^−1^) of the plant extract and lattice (455) indicated successful biosynthesis of BR- -NPs. Compared with the stem bark extract, 40 ng/dL dose of BR- -NPs led to a reduction in adipogenesis and an increase in mitochondrial biogenesis-related gene expressions, *adiponectin-R1*, *PPARγC1α*, *UCP-1*, and *PRDM16*, in maturing-adipocytes. This confirmed the intracellular uptake, bioavailability, and bio-efficiency of BR-TiO_2_-NPs. The lipid-lowering capacity of BR-TiO_2_-NPs effectively inhibited the metabolic inflammation-related gene markers, *IL-6*, *TNF-α*, *LTB4-R*, and *Nf-κb*. Further, BR-TiO_2_-NPs stimulating mitochondrial thermogenesis capacity was proven by the significantly enhanced CREB-1 and AMPK protein levels in adipocytes. In conclusion, BR-TiO_2_-NPs effectively inhibited lipid accumulation and proinflammatory adipokine levels in maturing adipocytes; it may help to overcome obesity-associated comorbidities.

## 1. Introduction

Nanotechnology is emerging rapidly with the development of nanosized materials, which have potential biomedical applications, especially in screening and preventing diseases. Nanoparticles of polymers, metals, and ceramides are included in modern drugs, which produce an enhanced activity against pathogens and diseases [1]. Nanoparticles are particulate materials possessing high impact that links higher to smaller size molecules; it comprises one dimension and less than 100 nm size [2]. The fabrication of nanoparticles by physical or chemical methods requires a high temperature, high pressure, and expensive chemicals that have toxic effects. Nevertheless, the synthesis of plant-based nanoparticles is an easier, biocompatible, and environmentally safe method, which reduces the eco-toxicity and lowers the energy waste associated with using chemicals [3].

The green synthesis of nanoparticles (NPs) is majorly preferred and depends on the biological reduction of metal ions by plants, bacteria, etc., to yield NPs with a uniform shape, size, and higher stability [4]. Metal oxide nanostructures or nanoparticles have been consecutively fabricated, evaluated, and used in commercial and medical applications for two decades [5]. In this way, titanium, silver, and zinc metals were used to design metallic NPs via synthetic or natural methods to reinforce pharmacological and medical applications [6]. Nanomaterials have a vast surface area, strong absorptivity, and high bioavailability, especially the good targeting properties and adjustable release rate, which might benefit the diagnosis and treatment of obesity and obesity-related diseases [7]. In this regard, manganese tetroxide nanoparticles (MnNPs, around 250 nm) were integrated into electrospun short fibers (SF@Rsg-Mn) and used to treat a diet-induced obesity mouse model; these nanoparticles led to weight loss by reducing fat, improvement in lipid metabolism, and a decrease in adverse effects on other tissues [8]. Green synthesized metal oxide nanomaterials possess a high oxidative capacity and mitochondrial potential in “in vitro” and “in vivo” models [9]. A previous study reported that TiO_2_-NPs interfere with epidermal growth factor receptor (EGFR) signaling cascade, inducing ROS-mediated cytotoxicity and genotoxicity as central underlying molecular mechanisms that lead to cell apoptosis in malignant cells, compared to neighboring physiological cells [10]. However, small-sized TiO_2_ nanoparticles have certain limitations in that they can mediate immune toxicity in rat pulmonary alveolar macrophages [11].

NPs with specific reduction capacity and levels are considered as new and appropriate elicitors for in vitro production and increasing the biosynthesis of secondary metabolites to use in pharmacological applications [9]. In this study, titanium oxide nanoparticles were prepared by reducing titanium ions with *Bauhinia rufescens* stem bark methanol extract. Then, the prepared nanoparticles were used in anti-obesity cell culture experiments. It is reported that *B. rufescens* contains phytochemical compounds, such as tannin, flavonoids, sterols, terpenes, saponins, polyphenolics, and tetracyclic compounds [12,13]. Therefore, *B. rufescens* is traditionally used to treat fibrosis, eye diseases, mycosis, gingivitis, diabetes, gout, and diarrhea. Among prominent NPs with demonstrations, both in vitro and in vivo, titanium oxide nanoparticles (TiO_2_-NPs) show unique surface chemistry and morphologies (e.g., sizes and shapes). They display good biocompatibility and exert inherent biological activities (e.g., efficient antimicrobial and antitumor properties) with weak side effects and low eco-toxicity [4]. Moreover, TiO_2_-NPs prepared using *Moringa oleifera* leaves were found to possess a potential wound healing activity [14]. Certainly, the mechanistic approach of metal NPs in cancer cells has been explored, which activates the apoptotic pathway through ROS production and subsequent anti-angiogenic, antiproliferative, and antitumor effects [10]. Compaore et al. [15] have found that the phenolics present in *B. rufescens* can enhance antioxidant potential via inhibition of xanthine oxidase and lipoxygenase enzymes. To our knowledge, green synthesized titanium oxide nanoparticles have rarely been previously studied in metabolic disorder models, especially those related to lipid accumulation. The present study aims to evaluate the potential of the fabricated TiO_2_/*Bauhinia rufescens* nanoparticles on inhibiting lipid accumulation and adipokine secretion in maturing adipocytes.

## 2. Results and Discussion

### 2.1. GC-MS Analysis

GC-MS analysis of *B. rufescens* stem bark methanol extract (BRME) revealed phytochemicals (Table 1, Figure S1) with considerable amounts, such as tridecanoic acid, 4,8,12-trimethyl-, methyl ester (53.10% of the total peak area), 2,3-Dihydroindole-4-ol-2-one, 1H-Purin-6-amine, N-methyl-, and 3-Methylpyridazine (24.85% of the total peak area), 2,4,6-Cycloheptatrien-1-one (7.58% of the total peak area). The pyridazine core found in 3-Methylpyridazine is an important structural hallmark of some active compounds with pharmacological potentials. Synthetic compounds containing pyridazine fractions are found to inhibit prostaglandin or cyclooxygenase (COX-I and COX-II) enzymes, platelet cAMP phosphodiesterase, and thromboxane A2 synthase. They also possess anti-inflammatory and in vitro antibacterial, antifungal and anticancer activities [16,17]. 2,4,6-Cycloheptatrien-1-one (Tropone), tropolones, and α-hydroxytropolones and their derivatives are members of the troponoids class. They were observed to have antibacterial, antifungal, insecticidal, antimalarial, antitumor, anti-ischemic, and iron-chelating activities and inhibitory activity against polyphenol oxidase as well [18,19]. Ethyl tridecanoate, a carboxylic acid ester, was scrutinized for anti-diabetic activity and found to suppress inflammations in diabetic rats [20]. Spirocyclic 2-Coumaranone derivatives have pharmacological activities against different biological targets [21,22]. The synthesized derivative, 4-[(butylsulfinyl)methyl]-1,2-benzenediol, an analogue of the natural compound 1,2-benzenediol, 3,5-bis(1,1-dimethylethyl), has an anti-inflammatory effect on lipopolysaccharide (LPS)-stimulated BV2 microglia [23]. Synthetic derivatives of 1,2-Benzisothiazol-3-amine, which belong to the compounds containing the isothiazole nucleus, are acknowledged as antimicrobial, antiproliferative, and anti-inflammatory agents [24].

### 2.2. Characterization of TiO_2_ Nanoparticles

The FT-IR spectra analysis was performed to identify chemical groups that enhanced titanium oxide nanoparticles’ bioactivity. BRME showed a broad, strong peak at 3405 cm^−1^, representing the O–H stretching vibration of the phenolics or carboxylic acids. This peak was blue-shifted with decreased intensity to 3402 and 3369 cm^−1^ in 3 and 6 mM BR-TiO_2_-NPs (Figure S2b), suggesting an interaction of O–H groups with titanium to form nanoparticles [29]. The peaks at 2925 and 2224 cm^−1^ were related to the C–H stretching vibrations of −CH_2_ groups of alkanes and -C ≡ C- asymmetrical stretching vibrations of alkynes [30]. BRME showed a strong absorption peak at 1615 cm^−1^, which correlated to asymmetrical stretching vibrations of carboxylic groups and nitro compounds [31,32]. The strong bands at 1607–1606 cm^−1^ observed in the BR-TiO_2_-NPs spectrum were ascribed to the binding of (NH)-C=O to TiO_2_. The band at 1440–1520 cm^−1^ in TiO_2_ and BRME was related to amide II [33]. Peaks at 1068–1070 cm^−1^ in BRME and BR-TiO_2_-NPs corresponded to C–N stretching vibrations of aromatic and aliphatic amines [32,34]. Further, the other two peaks located at 834 (minor) and 532 cm^−1^ (major) were found in BRME, related to the aromatic C–H bending of phenolics [35] and aromatic nitrile vibrations, respectively [29]. TiO_2_ showed a major peak at 455 cm^−1^, assigned to the O–Ti–O lattice stretching vibrations [30,36]; it was shifted to 494 and 466 cm^−1^ after the synthesis of BR-TiO_2_-NPs. Moreover, peaks at 1628 and 3405 cm^−1^ were noticed in the TiO_2_ spectrum, related to the O–H bending mode of water and/or surface hydroxides, and hydrogen bonding and O–H stretching from surface absorbed water [37].

Figure S2c shows the UV-vis absorption spectra of BRME, TiO_2_, and BR-TiO_2_-NPs. The absorption peak of TiO_2_ was below 400 nm. The UV-vis absorption peak of 6 mM BR-TiO_2_-NPs was red-shifted compared with the absorption band of TiO_2_, while that of 3 mM BR-TiO_2_-NPs was blue-shifted, suggesting the activation of the optical properties of TiO_2_ by BRME [38], as seen in the transformation of the basic solution color into yellowish (Figure S2a). Further, the 6 mM BR-TiO_2_-NPs had a stronger UV absorption intensity than 3 Mm BR-TiO_2_-NPs, indicating more semiconductor scavenging holes could be produced, resulting in higher photocatalytic activity [38].

**Figure 1:**
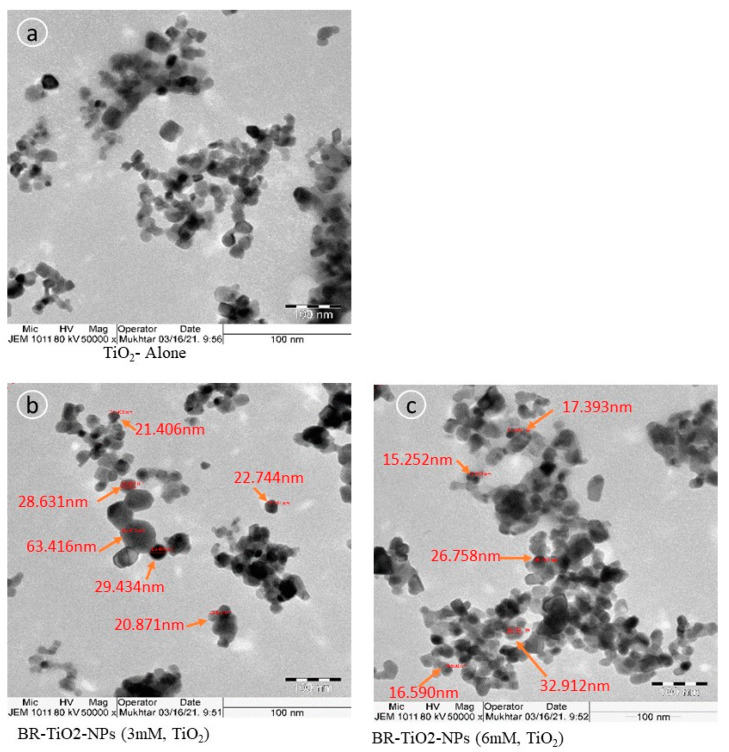
TEM images of TiO_2_ (**a**), 3 mM BR-TiO_2_-NPs (**b**), and 6 mM BR-TiO_2_-NPs (**c**). TEM images show tetragonal crystallites with diameters of 21.406 to 63.416 nm in 3 mM BR-TiO_2_-NPs and 15.252 to 32.912 nm in 6 mM BR-TiO_2_-NPs.

XRD patterns of TiO_2_ and *B. rufescens* stem-bark extract-loaded TiO_2_ (3 and 6 mM) nanoparticles revealed 13 similar characteristic diffraction peaks (Figure S3). TiO_2_ had a major diffraction peak at 2θ = 25.20°, related to (101) orientation plane, and another four peaks at 2θ = 37.67° (004), 48.00° (200), 53.77° (105), and 54.91° (211). These peaks confirmed the tetragonal anatase phase of TiO_2_ (JCPDS card no. 01-078-2486). The peaks at 2θ = 62.73° (204), 68.63° (116), 70.38° (220), 74.93° (215), and 82.64° (224) were also characterized as the anatase crystal phase (JCPDS no. card no. 01-078-2486). Moreover, the peaks at 2θ = 27.32° (110), 36.01° (101), and 41.23° (111) were assigned to the tetragonal rutile crystallographic phase (JCPDS card no. 21-1276). Similar phase orientation planes were observed in 3 and 6 mM BR-TiO_2_-NPs, but with slight deviations in 2θ positions, confirming the anatase crystallographic form of TiO_2_. These results agreed with the findings reported earlier on studying the biosynthesis of TiO_2_ nanoparticles [38,39,40]. The size distribution of 3 and 6 mM BR-TiO_2_-NPs was observed in the range of 30–500 d.nm (Figure S4). TEM images showed tetragonal crystallites with diameters of 35.28 and 15.25 nm for 3 and 6 mM BR-TiO_2_-NPs, respectively (Figure 1). The 6 mM BR-TiO_2_-NPs sample had good dispersion and lower particle size, so it was selected to carry out the biological experiments.

BR-TiO_2_-NPs having good functionality and stability were prepared. The bioactivity of fabricated TiO_2_ nanoparticles, composed mainly of the anatase crystal form and traces of rutile form, is boosted by carboxylic acids, peptides, and alcohols (UV-vis spectra; FT-IR spectra). These organic compounds can act as hole scavengers (carboxylic acid and amide/peptide groups) during the trapping of metastable photo-induced electrons by the nanoparticles [41]; thus, they could impart negative repulsive forces and lead to nanoparticles stability. Moreover, the TiO_2_ anatase phase has advantages of high photoactivity, lower cost, stability, and negative conduction band potential, so it is the most efficient photocatalyst frequently used in various fields [42]. Photocatalysts composed of a mixture of rutile and anatase phases are found to exhibit boosted photoactivity relative to the single-phase TiO_2_ [43].

### 2.3. Cytotoxicity

**Figure 2:**
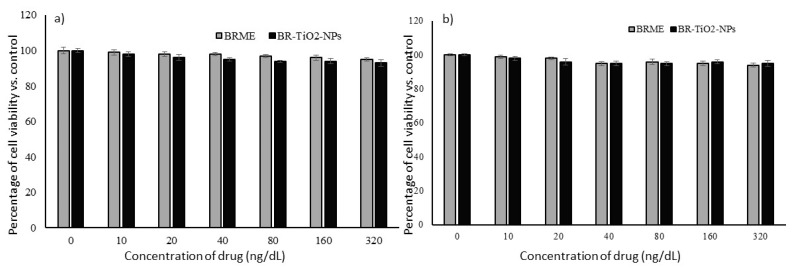
Effect of BRME and BR-TiO_2_-NPs treatments on cellular proliferation levels in hMSCs (**a**) and adipocytes (**b**) after 48 h. Each value is a mean ± SD (*n* = 6).

Identifying new agents (extract/nanoparticles) for anti-adipogenesis or lipid metabolism modulation potential requires primarily screening for biological safety via cell proliferation or viability inhibition potential. An MTT assay has been used to determine the cell proliferation or inhibition effects of BRME and BR-TiO_2_-NPs. Such increasing concentrations of BRME and BR-TiO_2_-NPs were selected and treated for up to 48 h with hMSCs and adipocytes, respectively. The results confirmed that in hMSCs, BR-TiO_2_-NPs produced a minimal of 7% inhibition and BRME showed 5% of cell viability inhibition only in the highest dose (320 ng/dL) after 48 h (Figure 2). In preadipocytes, 5% and 6% of cell inhibition were detected in BR-TiO_2_-NPs and BRME, respectively (Figure 2a,b), pinpointing the insignificant cell death that did not reach IC_10_ levels. The MTT assay revealed that the cellular inhibition produced by BR-TiO_2_-NPs or BRME was very small and insignificant, confirming their cytocompatibility of BR-TiO_2_-NPs and BRME with hMSCs or adipocytes. The above findings support the statement that TiO_2_ is the most promising material in the group of metal oxides; so, it has been approved by the US FDA for use in human foods, drugs, and food contact materials [27].

### 2.4. Biosafety of BR-TiO_2_-NPs in hMSCs

**Figure 3:**
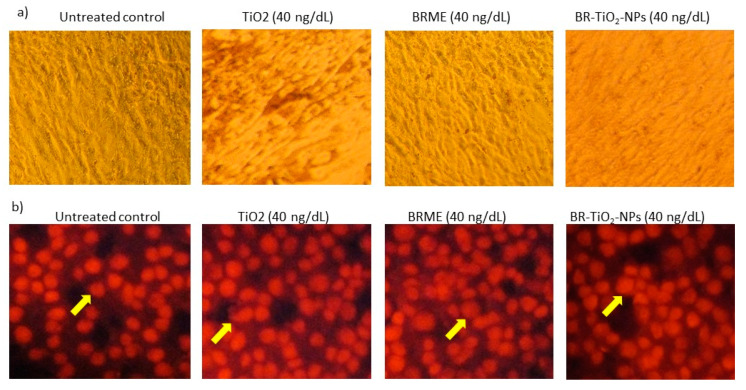
hMSCs cell morphology assay for the biosafety. (**a**) Light microscopy images and (**b**) Propidium iodide staining images of the control, and adipocytes treated with TiO_2_, BRME, and BR-TiO_2_-NPs for 48 h.

**Figure 4:**
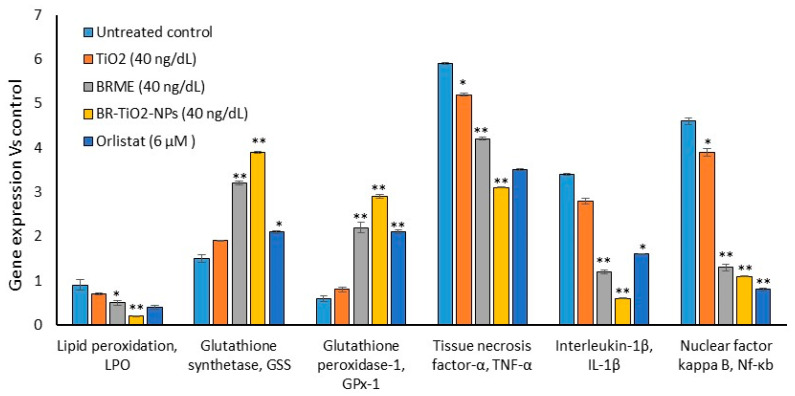
Oxidative stress and antioxidant-related gene expressions in the untreated control, TiO_2_, BRME, BR-TiO_2_-NPs, and orlistat-treated hMSC after 48 h. Each value is a mean ± SD (*n* = 6). * Significant at *p* ≤ 0.05 and ** highly significant at *p* ≤ 0.001, by comparison with the untreated control.

Nuclear staining (PI) of hMSCs after TiO_2_, BRME, and BR-TiO_2_-NPs treatments for 48 h did not show any nuclear damage or pyknosis (Figure 3). Most interestingly, PI staining revealed spherical-shaped nuclei in hMSCs treated with BR-TiO_2_-NPs. Previously, Muhammad and Sirat [44] have identified bioactive molecules, such as cyanoglucoside, menisdaurin, and oxepin present in *B. rufescens* stem bark, which decreased proinflammatory cytokines, COX-2, and increased anti-inflammatory conditions. Furthermore, TiO_2_ was noticed to increase the activity of the antioxidant enzyme catalase, stimulate glutathione transferase, and inhibit bacterial growth [33]. Tridecanoic acid, 4,8,12-trimethyl-, methyl ester was the major component of BRME, which has a derivative named 13-((2R)-6-hydroxy-2,5,7,8-tetramethylchroman-2-yl)-2,6,10-trimethyltridecanoic acid (α-T-13′-COOH). This derivative, which can be synthesized from α-tocopherol in a human liver-on-chip, is capable of inhibiting 5-lipoxygenase activity in human leukocytes and efficiently suppresses inflammation and bronchial hyper-reactivity in mouse models of peritonitis and asthma [25]. N6-methyladenine (24.85%) was reported to have a potential epigenetic role for ALKBH1–6 mA regulation in hypertension development, diagnosis, and treatment, as well as DNA damage repair [27,45]. In the present study, TiO_2_-treated hMSCs showed a decrease in oxidative stress, proinflammatory markers, and an increase in the antioxidant gene expressions compared with those in the untreated control. Collectively, the availability of bioactive compounds in *B. rufescens* stem bark that enhanced the antioxidant capacity via quenching the oxidative stress was observed in BRME treated hMSCs. *B. rufescens* stem bark methanol extract loaded with TiO_2_ generated functionalized BR-TiO_2_-NPs, with increased bioavailability and bio-efficiency than the stem bark extract. As seen, BR-TiO_2_-NPs enhanced glutathione synthetase (*GSS*) and glutathione peroxidase-1 (*GPX-1*) expressions and decreased proinflammatory cytokine expressions with no signs of cytotoxicity (Figure 4). The reference drug orlistat is an inhibitor of lipase enzymes that can arrest the hydrolysis of triglycerides. In the present study, hMSCs were used as a cell model to differentiate into adipocytes, and further, the inhibition of lipogenesis in the adipocytes by BR-TiO_2_-NPs was analyzed. In this view, the oxidative stress and the inflammatory cytokine generation capacity of orlistat were analyzed in hMSCs, comparatively, no negative effects were noticed. Most notably, orlistat treatment significantly reduced the expression level of peroxidative (*LPO*), proinflammatory cytokine (*TNF-α*, *IL-1β*, *Nf-κB*) expression and increased antioxidant genes (*GSS*, *GPX-1*) when compared to untreated control hMSCs (Figure 4).

### 2.5. Dose Determination Based on Lipid Accumulation Inhibition Potential by BR-TiO_2_-NPs

**Figure 5:**
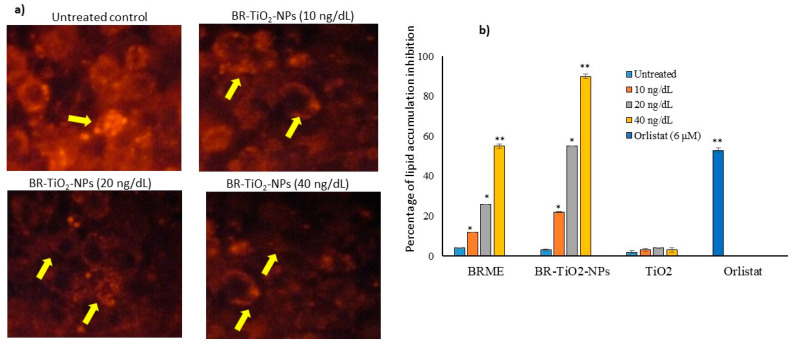
Effective dose determination for BR-TiO_2_-NPs by the lipid accumulation inhibition potential assay on adipocytes maturation after 14 days. Images of lipid accumulation by Nile red staining (**a**) and the lipid inhibition percentage as quantified after oil red’O staining (**b**). Each value is a mean ± SD (*n* = 6). * Significant at *p* ≤ 0.05 and ** highly significant at *p* ≤ 0.001, by comparison with the untreated control.

**Figure 6:**
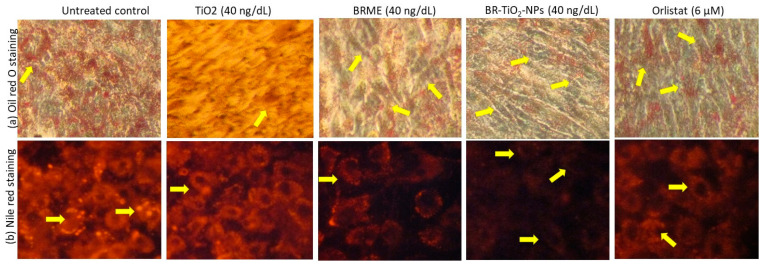
Oil red’O (**a**) and Nile red (**b**) analyses for the lipid accumulation inhibition potential in the untreated maturing adipocytes (control) and adipocytes treated with TiO_2_, BRME, and BR-TiO_2_-NPs for 14 days.

Initially, preadipocytes differentiation was confirmed by morphological analysis. Then, 10, 20, and 40 ng/dL doses of TiO_2_, BRME, and BR-TiO_2_-NPs were selected to assess lipid accumulation inhibition potential as per the experimental protocol. After 14 days, images of Nile red staining (Figure 5a) showed a significant 90% reduction of lipid droplets after maturing adipocytes treated with 40 ng/dL BR-TiO_2_-NPs, compared with the untreated control. The results of quantification of oil red’O staining (oil red’O images not presented) showed that BRME significantly decreased (*p* ≤ 0.001) the lipid accumulation by 55% in 40 ng/dL dose and 26% in 20 ng/dL when compared with the untreated control (Figure 5b). Moreover, a 40 ng/dL dose of TiO_2_ decreased 13% of the lipid accumulation, with a non-significant magnitude. The lipid accumulation inhibitory effect was significantly higher in 40 ng/dL of BRME or BR-TiO_2_-NPs compared with lower treatment doses; further, the lipid inhibition potential of BR-TiO_2_-NPs was significantly higher than BRME. BRME showed a similar decrease in lipid accumulation as the reference drug (6 μM of orlistat, 53% reduction). While the same dose of BR-TiO_2_-NPs inhibited 37% more lipid accumulation than 6 μM orlistat. The oil red’O and Nile red staining images of adipocytes treated with BR-TiO_2_-NPs (Figure 6a,b) showing the inhibition of lipid accumulation and hypertrophic adipocytes have been confirmed by the appearance of linear and spindle-shaped matured adipocytes, compared to untreated control or BRME or TiO_2_. This effect might be due to the enhanced internalization and cellular uptake of green synthesized BR-TiO_2_-NPs, which could stimulate nanoparticle lipolytic potentials via the surfactant bioactive compounds. Kanoujia et al. [46] found that the encapsulation of atorvastatin in soy protein isolate (SPI) and whey protein concentrate (WPC) nanoparticles (NPs) increased the cholesterol-lowering capacity of atorvastatin. Moreover, Joyce et al. [47] have found the encapsulation of rifampicin in mesoporous silica nanoparticles increased the cellular uptake and antibacterial activity.

### 2.6. Mitochondrial Function and Oxidative Capacity

**Figure 7:**
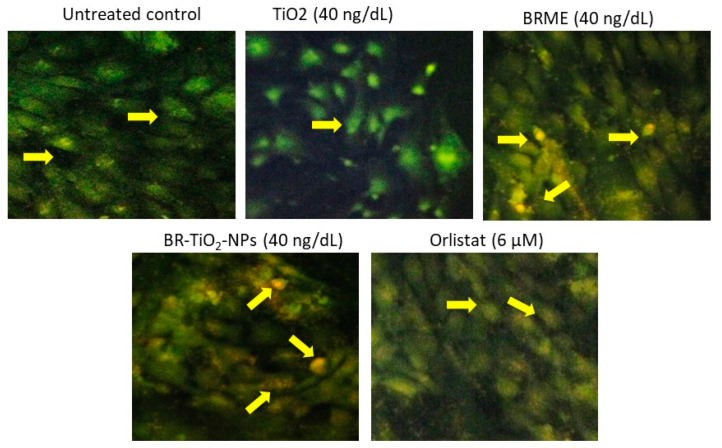
JC-1 staining analysis for mitochondrial membrane potential in the control adipocytes and the adipocytes treated with TiO_2_, BRME, and BR-TiO_2_-NPs for 14 days.

Figure 7 shows the JC-1 staining images of the untreated control and adipocytes treated with TiO_2_, BRME, and BR-TiO_2_-NPs (40 ng/dL). The JC-1 staining images displayed red and green signals, corresponding to J-aggregates and monomeric forms, respectively. We revealed that a 40 ng/dL dose of BRME or BR-TiO_2_-NPs resulted in high J-aggregates, representing the potential of mitochondrial efficiency on thermogenesis. TiO_2_ and orlistat treated maturing adipocytes showed very few J-aggregates, illustrating a lower mitochondrial potential.

### 2.7. Adipogenesis and Lipolysis Related Gene Expressions

**Figure 8:**
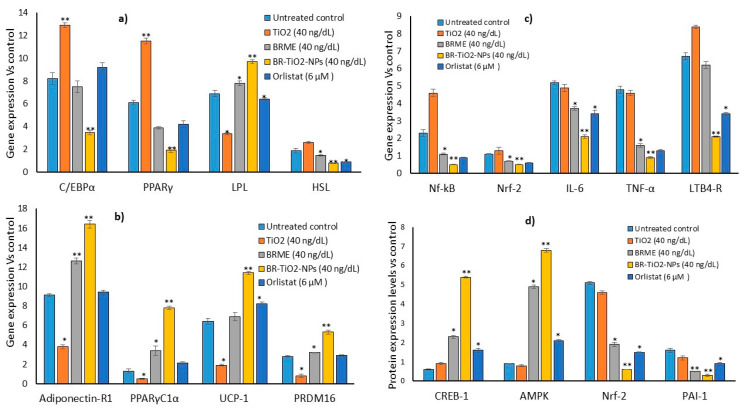
Adipogenesis (**a**), mitochondrial thermogenesis (**b**), and adipocytokine (**c**)-related gene expression levels and mitochondrial oxidative capacity signaling protein (**d**) levels in the untreated control adipocytes and the adipocytes treated with TiO_2_, BRME, and BR-TiO_2_-NPs for 14 days. Each value is a mean ± SD (*n* = 6). * Significant at *p* ≤ 0.05 and ** highly significant at *p* ≤ 0.001, by comparison with the untreated control.

Intensive studies targeting obesity have been conducted using plant-based gold nanoparticles [48], but the use of titanium nanoparticles to treat obesity is rarely covered. However, the synthesis of titanium oxide nanoparticles from different biological sources (plants, microbes, and related bio-products) and their biological application includes photodynamic cancer treatment and antimicrobial therapies, have been reported earlier [3]. In this study, we explored the lipid-lowering effects of BR-TiO_2_-NPs prepared by using *B. rufescens* stem bark methanol extract (BRME). We noticed a decrease in mRNA expression levels adipogenesis associated traits, such as *C/EBPα* and *PPAR*, which were decreased two-fold after BR-TiO_2_-NPs treatment (Figure 8a). During adipogenesis, the *C/EBPα* expression is found to be a very early event and stimulates the downstream upregulated *PPAR-γ*. *C/EBPα* and *PPAR-γ* are the central transcriptional regulators of adipogenesis, and most of the adipocyte functional proteins’ synthesis was stimulated by these regulators [49]. The protein expression of *PPAR* and *C/EBP* was decreased in adipocytes treated with capsaicin, genistein, berberine, and EGCG [49]. Apart from inhibiting adipogenesis, stimulating lipolysis in adipocytes might be more beneficial in controlling insulin resistance. Flavonoids genistein, daidzein, coumestrol, and zearalenone stimulate lipolysis; quercetin, luteolin, and fisetin caused an increase in lipolysis, which was synergistic with epinephrine in rat adipocytes [49].

Functional modifications of metal oxide can aid the intracellular uptake and bioavailability of BR-TiO_2_NPs into adipocytes and stimulate the lipolytic gene expression pathway. This was confirmed by an increased expression of mitochondrial thermogenesis-associated genes, such as *adiponectin-R1*, *PPARγC1α*, *UCP-1*, and *PRDM16* (Figure 8b). In adipocytes, the cAMP–PKA pathway stimulates the level of *PGC1α,* which activates the transcription of thermogenic genes, such as *PRDM16* and *UCP1*, and increases *CREB* levels [50,51]. Kang et al. [52] observed that punicalagin had decreased the levels of the Nf-κb signaling pathway associated with the inhibition of obesity-related inflammatory response. It was acknowledged that the proanthocyanidins in grape seeds stimulated long-term lipolysis by the activation of the β3-adrenergic receptor and the ERK signaling pathway, which increased cAMP and PKA in 3T3-L1 adipocytes [53].

Failure in lipolysis can result in an excessive accumulation of triglycerides in adipocytes, ending with hypertrophic adipocytes and metabolic stress, leading to vascular complications [54]. In addition, the anti-obesity effect and inhibition of obesity-associated inflammatory response in adipocytes were identified with decreased levels of *NF-kB* and *TNF-α* (Figure 8c). In this study, we observed that BR-TiO_2_-NPs effectively reduced lipid accumulation and increased mitochondrial thermogenesis, as proven by enhanced thermogenesis-associated gene expressions. This effectively reduced the amount of hypertrophic adipocytes and, subsequently, inflammatory cytokine expressions, such as *IL-6*, *TNF-α*, *LTB4-R*, *Nrf-2*, and *Nf-κb*, in adipocytes, were reduced. BR-TiO_2_-NPs effectively decreased proinflammatory cytokines in hMSCs (Figure 4). In adipocytes, hyperlipidemic conditions generated oxidative stress, and inflammatory cytokines were decreased (Figure 8), showing antiperoxidative and anti-inflammatory effects. In this context, Katiyar et al. [55] found that titanium nanoparticles led to a decrease in malondialdehyde levels and antiperoxidative effects. Previously, we have reported that *Ziziphus spina-christi* (Jujube) root methanol extract-loaded functionalized silver nanoparticle (ZS-Ag-NPs) can effectively control adipocyte maturation and adipokine secretion levels [56]. It was reported that titanium oxide nanoparticles have antioxidant and antiperoxidative potentials [32].

Hypertrophic adipocytes attract macrophages and secrete adipocytokines responsible for metabolic inflammations and secondary complications. The results of the related gene expression levels and mitochondrial oxidative capacity-signaling protein levels in the control, TiO_2_, BRME, and BR-TiO_2_-NPs-treated maturing adipocytes after 14 days are shown in Figure 8d. BR-TiO_2_-NPs significantly increased *CREB-1* and *AMPK* and decreased *Nrf-2* and *PAI-1* factors in maturing adipocytes. The stimulation of *CREB-1* and *AMPK* protein activation in adipocytes became more challenging because of the low internalization and extracellular digestion of the drug (Figure 8d). Green synthesis of BRME-loaded TiO_2_ nanoparticles possess functional groups (-OH, -C≡C-, and carboxylic and nitro groups) of phytochemicals detected in the BRME, which interact with titanium to form stable and functionalized nanoparticles. High lipid levels stimulate oxidative stress, further initiating a series of toxic oxidative reactions accompanied by the downregulation of the nuclear erythroid-related factor 2 (*Nrf-2*) genes [57]. *Nrf-2* is considered the main regulator of antioxidant response. *Nrf-2* activation decreases oxidative stress and helps manage neurodegenerative diseases through the upregulation of antioxidants, inhibition of inflammation, augmenting mitochondrial function, and protein homeostasis [58]. In this context, the green synthesis of titanium dioxide nanoparticles using *Psidium guajava* extract was found to possess antibacterial and antioxidant properties [59]. Previously, we have reported that the synthesis of basil seed-loaded solid lipid nanoparticles enhanced the bioavailability and lipolytic potential and decreased the adipocytokine potential in maturing adipocytes [32].

## 3. Materials and Methods

### 3.1. Preparation of Bauhinia rufescens (Lam.) (kulkul) Stem Bark Methanol Extract

*Bauhinia rufescens* (Lam.) stem barks were obtained from Darfur-Sudan and identified by a taxonomist in King Saud University, Riyadh (a specimen sample is kept in the Department Herbarium). The shade-dried stem barks were crushed and suspended in 95% methanol at a solid-to-solvent ratio of 1:10 in a conical flask. After wrapping with an aluminum foil, the flask was shaken for 6 h using a Wrist Action shaker (Burrell Scientific, Pittsburgh, PA, USA). The extractive was filtered (Whatman No. 1 filter paper) and then concentrated in vacuo using a rotary evaporator (HAHNVAPOR, HS-2005, Hahn Shin Scientific, Gimpo-si, Korea). The concentrated stem bark (41.1 mg/mL) was kept for further use.

### 3.2. GC-MS Analysis of the Extract

The GC-MS composition of *Bauhinia rufescens* stem bark methanol extract (BRME) was analyzed using an Agilent 7890A (Agilent Technologies, Santa Clara, CA, USA) gas chromatography (GC) coupled with a 5975C inert mass-spectrometer (MSD). The system was equipped with a DB-5MS GC column (30 m length, 0.25 mm inner diameter, and 0.25 µm film thickness), a Triple-Axis detector (MSD), and a 7693 automated liquid sampler. One milliliter of the extract was filtered through a 2 µm membrane filter. An aliquot (1 µL) of the extract was injected into the system. The injection temperature was 280 °C, and the column temperature was 300 °C. The carrier gas was helium, with a flow rate of 1 mL/min. The electron ionization energy was 70 eV.

### 3.3. Synthesis of Titanium Oxide Nanoparticles

*Bauhinia rufescens* stem bark methanol extract (BRME)-loaded titanium nanoparticles (BR-TiO_2_-NPs) were synthesized by drop-wise addition of 10 mL of the BRME extract to 90 mL of TiO_2_ solutions (concentrations: 3 mM and 6 mM) in the dark at 50 °C, with continuous stirring (200 rpm, 90 min). Then, the pH of the reaction mixtures was adjusted to 1.5, and the mixtures were left to react at 50 °C for 5 h. A yellowish-brown color was developed, indicating the formation of BR-TiO_2_-NPs. The nanoparticle solutions were centrifuged (12,000 rpm, 20 min) and dried at 60 °C for 24 h. The dried nanoparticles were milled and kept until use. Classic TiO_2_ nanoparticles, to serve as a control, were simultaneously prepared by the same protocol using a 95% methanol solution instead of BRME.

### 3.4. Characterization of Titanium Oxide Nanoparticles

To check the formation of BR-TiO_2_-NPs, the functionalization of TiO_2_ was examined by a UV-visible spectrophotometer (UV-2450 double-beam, Shimadzu, Tokyo, Japan). The UV-visible spectra of the BRME, TiO_2_, and BR-TiO_2_-NPs (3 and 6 mM) were measured at a wavelength range of 200–800 nm. The crystalline phase analysis of the synthesized nanoparticles was performed by measuring X-ray powder diffraction (XRD) patterns using a diffractometer (Bruker D8 Advance) equipped with a Cu-Kα radiation source (λ = 1.54 nm; 40 kV; 40 mA) and a diffracted beam monochromator. The scattered radiations were detected in the angular range of 10–90° (2θ) with a scan rate of 0.02°. Diffraction patterns of the stem bark methanol extract, TiO_2_, and BR-TiO_2_-NPs colloids were compared with the JCPDS card database. The functional groups of the BR-TiO_2_-NPs and BR-TiO_2_-NPs were analyzed by using a Nicolet 6700 Fourier-transform infrared (FT-IR) spectrometer (Waltham, MA, USA) at a wavenumber ranging between 500 and 4000 cm^−1^. Morphological images of samples were taken by a transmission electron microscope (TEM) (JEM-1011, JEOL Ltd., Tokyo, Japan) working at an acceleration voltage of 160 kV. The size distribution by the intensity of BR-TiO_2_-NPs was determined by using a Zetasizer (HT Laser, ZEN3600 Malvern, Nano series, Instruments, Malvern, UK).

Before starting the cellular experiments, nanoparticle powder samples were sterilized using UV radiation for 10 min to eliminate any microbial contaminations that could happen during storage. All nanoparticle dilutions used were prepared freshly.

### 3.5. In Vitro Study in Maturing Adipocytes

#### 3.5.1. Chemicals

Human mesenchymal stem cells (hMSCs) were obtained from the American Type Culture Collection (ATCC, Manassas, VA, USA). Dulbecco’s modified Eagle medium (DMEM), trypsin, EDTA, and all cell culture materials were purchased from Gibco, Paisley, UK. Cell culture materials, such as fetal bovine serum and penicillin-streptomycin, were obtained from HyClone Laboratories, USA. ORO Oil red’O, Nile red, and MTT [3-(4,5-dimethylthiazol-2-yl)-2,5-diphenyltetrazolium bromide], were purchased from Sigma-Aldrich (St. Louis, MO, USA). Adipocyte differentiation factors, such as insulin, rosiglitazone, dexamethasone (DEX), and 3-isobutyl-1-methyl-xanthine (IBMX) were purchased from Sigma-Aldrich (St. Louis, MO, USA). The cytokine-analyzing ELISA array kits were obtained from Qiagen (MEH004A, Qiagen, Hilden, Germany). The cDNA synthesis kit and SYBR Green PCR Master Mix were purchased from Qiagen, Hilden, Germany. All other chemicals used to carry out molecular biology experiments were purchased from Sigma-Aldrich (St. Louis, MO, USA).

#### 3.5.2. Human Mesenchymal Stem Cells (hMSCs) Culture and Adipocyte Differentiation

hMSCs were cultured using Dulbecco’s modified Eagle medium (DMEM) containing 10% fetal bovine serum and 100 U/mL penicillin-streptomycin at 37 °C in a humidified 5% CO_2_ using an incubator. Cells were cultured in 24-well plates at a density of 2 × 10^4^ cells/well. The cells were grown, to reach 90% confluence, in DMEM/high glucose containing 10% FBS at 37 °C and 5% CO_2_ humidified air. Forty-eight hours after visual confluence (day 0), cells were replaced with adipocyte differentiation media (DMEM containing 10% FBS, 1 µM dexamethasone, 0.5 mM IBMX, and 10 μg/mL insulin) for the next three days. On Day 3, the cells were then cultured in adipogenesis maturation medium (DMEM containing 10% FBS and 10 μg/mL insulin) for two consecutive days. Subsequently, the cells were cultured in a maintenance medium (DMEM with 10% FBS) for six days, a fresh medium was replaced every two days. For all assays, cells cultured only in the maintenance medium were used as a control.

#### 3.5.3. Cytotoxicity Analysis

hMSCs were induced to differentiate into adipocytes in 96-well culture plates (1 × 10^4^ cells/well) and allowed to adhere overnight in DMEM. After discarding the medium, a culture medium containing BRME or 6 mM BR-TiO_2_-NPs (0, 10, 20, 40, 80, 160, and 320 ng/dL) was added to each well, and the cells were incubated for 24 to 48 h; untreated cells were used as controls. After the incubation, the cells were carefully washed with PBS, then a medium containing 5 mg/mL MTT (3-[4,5-dimethylthiazol-2-yl]-2,5-diphenyltetrazolium bromide) in DMEM was added to each plate well (i.e., 20 µL/well). The plates were then incubated at 37 °C for a further 4 h, a purple formazan precipitated. Next, the medium was removed, and the formazan was dissolved in 100 µL of DMSO. Then, the absorbance was read at 570 nm using a microplate reader (Thermo Scientific, Waltham, MA, USA). The cell proliferation (%) was calculated by the following equation: (absorbance of the sample/mean absorbance of the control) × 100.

#### 3.5.4. Propidium Iodide Staining for Nuclear Damage Analysis in hMSCs

The ability of the untreated control, BRME, TiO_2_, and BR-TiO_2_-NPs, on the stimulation of nuclear damage in hMSCs was quantified using propidium iodide (PI) (Sigma Chemicals, St. Louis, MO, USA). hMSCs were plated in a 24-well plate (1 × 10^4^/well) and allowed to confluence, and treated with BRME, TiO_2_, and BR-TiO_2_-NPs (40 ng/dL), then incubated for 48 h. Again, treated hMSCs were incubated with 5 μL of PI in the dark for 15 min at room temperature. After PI staining, cell images were captured by an inverted microscope (ZEISS Axio Vert.A1, Carl Zeiss Microscopy, LLC, White Plains, NY, USA) and analyzed to identify nuclear damages or condensation levels.

#### 3.5.5. Experimental Design for the Antiobesity Study

The differentiated adipocytes (3rd day) were treated with different concentrations (10, 20, and 40 ng/dL) of freshly prepared BRME, TiO_2_, BR-TiO_2_-NPs (6 mM), and orlistat (6 µM, reference drug) solutions. The working dilutions were prepared from the sample stock dispersion and added to 200 μL of the cell suspension in a 96-well plate or 500 μL of the cell suspension in a 24-well plate. Then, drug-treated maturing adipocytes were maintained for day 14; the media were replaced with the maintenance medium once in 3 days. The selection of the effective dose was based on the lipid accumulation inhibitory effect of BRME, TiO_2_, BR-TiO_2_-NPs after 14 days. In another set of experiments, the condition media of the untreated adipocytes and adipocytes treated with BRME, TiO_2_, BR-TiO_2_-NPs, and orlistat (6 µM) were collected on day 14.

#### 3.5.6. Oil Red’O and Nile Red Staining Analysis to Determine Lipogenesis Levels

Adherent differentiated preadipocytes were subsequently treated with 10, 20, and 40 ng/dL doses of freshly prepared BRME, TiO_2_, BR-TiO_2_-NPs, and orlistat (6 µM) in the desired medium (dose selected based on cytotoxicity analysis). Experimental cells were maintained for 14 days by changing with maintenance media once in 3 days. After 14 days, the cells were washed twice with PBS and fixed with 4% (*v*/*v*) paraformaldehyde for 1 h at room temperature. Thereafter, cells were washed with isopropanol 60% (*v*/*v*) and allowed to dry. Then, treated cells were stained with a filtered 0.5% oil red’O solution (*v*/*v*) (60% isopropanol and 40% water) for 1 h. After staining, the oil red’O staining solution was removed, then the plates were rinsed with distilled water 3 times and dried. The stained lipid droplets were viewed at 20× magnification on a microscope and photographed. After analyzing the microscopic images, the stained cells were allowed to dry overnight, and the oil stains were dissolved with isopropanol to measure the absorbance at 520 nm.

For the Nile red staining assay, a solution containing 5 mg of Nile red dissolved in 1 mL of 100% acetone was used. After 14 days of BRME, TiO_2_, BR-TiO_2_-NPs, and orlistat (6 µM) treatments, preadipocytes were fixed with formaldehyde, then stained with 200 μL of fluorescence Nile red (working solution: 6 μL of stock Nile red dissolved in 1 mL of 40% isopropanol) for 30 min at room temperature. The stained cells were analyzed using an inverted fluorescence microscope, and photographs were taken immediately using a fluorescent microscope.

#### 3.5.7. Mitochondrial Membrane Potential Using the JC-1 Staining Assay

Mitochondrial membrane potential (Δψm) was determined using the JC-1 staining assay, which exists in monomeric form predominantly in cells with depolarized mitochondrial membrane with green fluorescence signals. Cells with polarized mitochondria uptake JC-1 and aggregate predominantly with reddish-orange fluorescence. The untreated control, BRME, TiO_2_, BR-TiO_2_-NPs, and orlistat (6 µM)-treated cells were incubated with 5 mM of JC-1 for 15 min at 37 °C. Then, washed with the JC-1 washing solution, and the fluorescence signals were analyzed with a fluorescent microscope.

#### 3.5.8. Quantitative Polymerase Chain Reaction (qPCR) Analyses

Total RNA was isolated from the untreated control, BR, TiO_2_, BR-TiO_2_-NPs (40 ng/dL), and orlistat (6 µM)-treated maturing adipocytes and hMSCs. Immediately, the total RNA was utilized to synthesize cDNA using a Fastlane^®^ Cell cDNA kit and a semi-automative qPCR instrument (Applied Biosystems, Foster City, CA, USA). Adipogenesis and hypertrophy *C*/*EBPα* (CCAAT/enhancer-binding protein-α), *PPARγ* (peroxisome proliferator-activated receptor-gamma), *HSL* (hormone-sensitive lipase), *LPL* (lipoprotein lipase), *SREBP-1c* (sterol regulatory element-binding of protein-1c), and *FABP-4* (Fatty acid-binding protein 4)), fatty acid oxidation and energy metabolism (*Adiponectin-R1*, *PPARγC1α* (peroxisome proliferator-activated receptor-gamma coactivator 1 alpha), *UCP-1* (uncoupling protein-1), and *PRDM16* (PR domain-containing protein 16))-related mRNA levels in adipocytes were quantified. In hMSCs, mRNA levels of oxidative stress (*LPO* (lipid peroxidation), *GSS* (glutathione synthetase), *Gpx-1* (glutathione peroxidase-1), *TNF-α* (tissue necrosis factor-alpha), *IL-1β* (interleukin-1 beta), and *Nf-κb* (nuclear factor kappa B))were quantified against the reference gene, *β-actin*, according to the method reported earlier [60]. The amplification values (ΔCt) were calculated based on the difference between the Ct value of treated maturing adipocytes and the Ct value of the control. Gene expressions were plotted using the expression of the 2^−ΔΔCt^ value. Primer sequences used in the real-time polymerase chain reaction (RT-PCR) are shown in Table 2.

#### 3.5.9. Quantification of Protein Using ELISA

The amount of metabolic inflammation, insulin resistance, and fatty acid metabolism deregulating markers, such as *CREB-1* (Cyclic AMP responsive element binding protein-1), *AMPK* (AMP-activated protein kinase), *Nf-κb*, and *TNF-α* (in adipocytes), were analyzed in the untreated control, BR, TiO_2_, BR-TiO_2_-NPs (40 ng/dL), and orlistat (6 µM)-treated cells using high-sensitivity ELISA kits (Quantikine, R&D Systems, Minneapolis, MN, USA). This assay gives a measure of the total concentration of inflammatory mediator proteins, and the values were expressed as pg/mg protein.

### 3.6. Statistical Analysis

All experimental data were statistically evaluated using SPSS/28.5 software package. The data were analyzed by the one-way analysis of variance (ANOVA) and followed by Tukey’s multiple comparison test. All results were expressed as mean ± SD for six replications in each group. Significant differences between means were stated at *p* < 0.05.

## 4. Conclusions

NPs can be used as photocatalysts, pharmaceuticals, cosmetics, and sunscreens [61,62]. In the present study, using nanomolar concentrations of BR-TiO_2_-NPs might be considered as a physiologically safer dose if applied to animal or human models. Usage of nanomolar concentrations of TiO_2_ NPs may be beneficial to overcome the major complications and limitations of TiO_2_-NPs accumulation in the brain tissues. TiO_2_-NPs can be rapidly absorbed via many pathways, such as the olfactory nerve translocation, the placental barrier [63], and the blood–brain barrier [64]. The bioactive components present in *B. rufescens* stem methanol extract were effectively internalized into TiO_2_, which yields functionally-enhanced BR-TiO_2_-NPs. Further, the phytochemicals-loaded nanoparticles can get internalized easily and increase intracellular bioavailability. Intracellular availability of BR-TiO_2_-NPs effectively regulated cytoplasmic or mitochondria-mediated signaling pathways associated with lipolysis and fatty acid oxidation mechanism. The enhanced anti-lipogenic effect, followed by the inhibition of adipokine level in maturing adipocytes by BR-TiO_2_-NPs, was more significant than BRME or TiO_2_ treatments. Most of the bioactive molecules are metabolized extracellularly or structurally modified during the absorption process and lose their potential. Further, to assess the effects of the bioactive compounds under the physiological conditions, in vivo studies are crucial.

## Figures and Tables

**Table 1:** GC-MS analysis of phytochemicals in *B. rufescens* stem bark methanol extract.

No	RT (min)	Peak Area (%)	Compound Name	Molecular Formula	Molecular Weight (g/mol)	Compound Nature	Bioactivity
1	18.05	7.58	2,4,6-Cycloheptatrien-1-one (Tropone)	C_7_H_6_O	106.12	Cyclic aliphatic ketone	Antibacterial, antifungal, insecticidal, antimalarial, antitumor, anti-ischemic, iron chelating, and inhibitory activity against polyphenol oxidase activity [18,19].
			2-Coumaranone	C_8_H_6_O_2_	134.13	Benzofurn ketone	Spirocyclic 2-Coumaranone derivatives have pharmacological activities against different biological targets [21,22].
2	23.99	53.10	Tridecanoic acid, 4,8,12-trimethyl-, methyl ester	C_17_H_34_O_2_	270.5	Aliphatic ester	Derivatives have immune-regulatory and anti-inflammatory functions [20,25].
			(Methylthio)-acetonitrile	C_3_H_5_NS	87.15	Thionitriles	Not reported.
3	25.69	24.85	1H-Purin-6-amine, N-methyl-(N6-Methyladenine)	C_6_H_7_N_5_	149.15	Purine	Antiprotozoal agents. DNA damage repair agents [26,27].
			3-Methylpyridazine	C_5_H_6_N_2_	94.11	Heterocyclic organic compound	Derivatives have antimicrobial, anticancer, and anti-inflammatory activities [16,17,28].
4	34.86	3.98	9-Octadecenoic acid (Z)-, methyl ester (Methyl Oleate)	C_19_H_36_O_2_	296.50	Fatty acid ester	Not reported.
			9-Octadecenoic acid, methyl ester, (E)-(Methyl eliadate)	C_19_H_36_O_2_	296.50	Fatty acid ester	Not reported.
5	43.54	4.30	1,2-Benzisothiazol-3-amine tbdms	C_13_H_20_N_2_ SSi	264.46	Heterocyclic compound	Derivatives have antimicrobial, antiproliferative, and anti-inflammatory activities [24].
6	52.52	6.19	1,2-Benzenediol, 3,5-bis(1,1-dimethylethyl)-	C_14_H_22_O_2_	222.32	Phenols	Anti-inflammatory effects [23].

**Table 2:** Primer sequences used in the sybr-green-based real-time polymerase chain reaction (RT-PCR).

Primer	Forward Sequence (5′ to 3′)	Reverse Sequence (5′ to 3′)
*LPO*	CTGCCCTATGACAGCAAGAAGC	CGGTTATGCTCGCGGAGAAAGA
*GPX-1*	GTGCTCGGCTTCCCGTGCAAC	CTCGAAGAGCATGAAGTTGGGC
*GSS*	GGAACTCCAACAAGGGAGCA	TTCGGGGTCGGAAGACCTT
*TNF-α*	CTCTTCTGCCTGCTGCACTTTG	ATGGGCTACAGGCTTGTCACTC
*IL-1β*	CCACAGACCTTCCAGGAGAATG	GTGCAGTTCAGTGATCGTACAGG
*NF-κb*	GCGCTTCTCTGCCTTCCTTA	TCTTCAGGTTTGATGCCCCC
*C/EBPα*	CCGGGAGAACTCTAACTC	GATGTAGGCGCTGATGT
*PPARγ*	TCATAATGCCATCAGGTTTG	CTGGTCGATATCACTGGAG
*LPL*	AGGACCCCTGAAGACAG	GGCACCCAACTCTCATA
*HSL*	CCTCATGGCTCAACTCC	GGTTCTTGACTATGGGTGA
*Adiponectin-R1*	CTACTGTTGCAAGCTCTC C	CTTCACATCTTTCATGTACACC
*PPARγC_1_α*	CCCTGCCATTGTTAAGACC	TGCTGCTGTTCCTGTTTTC
*UCP-1*	AGGCTTCCAGTACCATTAGGT	CTGAGTGAGGCAAAGCTGATTT
*PRDM16*	CCCCACATTCCGCTGTGA	CTCGCAATCCTTGCACTCA
*NRF-2*	CACATCCAGTCAGAAACCAGTGG	GGAATGTCTGCGCCAAAAGCTG
*IL-6*	AGACAGCCACTCACCTCTTCAG	TTCTGCCAGTGCCTCTTTGCTG
*LTB4-R*	CCTGTGTCACTATGTCTGCGGA	ATCGCCTTGGTGCGTAGCTTCT
*β-Actin*	GATCTTGATCTTCATGGTGCTAGG	TTGTAACCAACTGGGACCATATGG

## Data Availability

All data can be obtained from the corresponding author upon request.

## Supplementary Materials

The following are available online, Figure S1: GC-MS analysis of phytochemical compounds of *B. rufescens* stem methanol extract. Figure S2: (a) Synthesis of 3 and 6 mM BR-TiO_2_-NPs (C,D), (b) FT-IR spectra, and (c) UV-absorption spectra. Figure S3: XRD patterns of TiO_2_ (a), 3 mM BR-TiO_2_-NPs (b), and 6 mM BR-TiO_2_-NPs (c). Figure S4: Size distribution of TO_2_ (a), 3 mM BR-TiO_2_-NPs (b), and 6 mM BR-TiO_2_-NPs (c). Correspondence and requests for materials should be addressed to A.E.A.Y.

## Sample Availability

Samples of the titanium oxide nanoparticle compounds are available from the authors.

## References

[1] Ikram M., Javed B., Hassan S.W.U., Satti S.H., Sarwer A., Raja N.I., Mashwani Z.-U. (2021). Therapeutic potential of biogenic titanium dioxide nanoparticles: A review on mechanistic approaches. Nanomedicine.

[2] Srinivasan M., Venkatesan M., Arumugam V., Natesan G., Saravanan N., Murugesan S., Ramachandran S., Ayyasamy R., Pugazhendhi A. (2019). Green synthesis and characterization of titanium dioxide nanoparticles (TiO_2_ NPs) using Sesbania grandiflora and evaluation of toxicity in zebrafish embryos. Process. Biochem..

[3] Nadeem M., Tungmunnithum D., Hano C., Abbasi B.H., Hashmi S.S., Ahmad W., Zahir A. (2018). The current trends in the green syntheses of titanium oxide nanoparticles and their applications. Green Chem. Lett. Rev..

[4] Iqbal H., Razzaq A., Uzair B., Ain N.U., Sajjad S., Althobaiti N.A., Albalawi A.E., Menaa B., Haroon M., Khan M. (2021). Breast Cancer Inhibition by Biosynthesized Titanium Dioxide Nanoparticles Is Comparable to Free Doxorubicin but Appeared Safer in BALB/c Mice. Materials.

[5] Batool A., Menaa F., Uzair B., Khan B.A., Menaa B. (2020). Progress and Prospects in Translating Nanobiotechnology in Medical Theranostics. Curr. Nanosci..

[6] Uzair B., Liaqat A., Iqbal H., Menaa B., Razzaq A., Thiripuranathar G., Rana N.F., Menaa F. (2020). Green and Cost-Effective Synthesis of Metallic Nanoparticles by Algae: Safe Methods for Translational Medicine. Bioengineering.

[7] Li J., Cha R., Luo H., Hao W., Zhang Y., Jiang X. (2019). Nanomaterials for the theranostics of obesity. Biomaterials.

[8] Ding Z., Chen M., Tao X., Liu Y., He J., Wang T., Li X. (2021). Synergistic Treatment of Obesity via Locally Promoting Beige Ad-ipogenesis and Antioxidative Defense in Adipose Tissues. ACS Biomater. Sci. Eng..

[9] Modarresi M., Chahardoli A., Karimi N., Chahardoli S. (2020). Variations of glaucine, quercetin and kaempferol contents in Nigella arvensis against Al_2_O_3_, NiO, and TiO_2_ nanoparticles. Heliyon.

[10] Liu R., Zhang X., Pu Y., Yin L., Li Y., Zhang X., Liang G., Li X., Zhang J. (2010). Small-sized titanium dioxide nanoparticles mediate immune toxicity in rat pulmonary alveolar macrophages in vivo. J. Nanosci. Nanotechnol..

[11] Aliyu A., Ibrahim M., Musa A., Ibrahim H., Abdulkadir I., Oyewale A. (2009). Evaluation of antioxidant activity of leaves extract of *Bauhinia rufescens* Lam. (Caesalpiniaceae). J. Med. Plant Res..

[12] Maillard M.P., Recio-Iglesias M.C., Saadou M., Stoeckli-Evans H., Hostettmann K. (1991). Novel Antifungal Tetracyclic Compounds from *Bauhinia rufescens* Lam.. Helv. Chim. Acta.

[13] Sivaranjani V., Philominathan P. (2016). Synthesize of Titanium dioxide nanoparticles using *Moringa oleifera* leaves and evaluation of wound healing activity. Wound Med..

[14] Kim H., Jeon D., Oh S., Nam K., Son S., Gye M.C., Shin I. (2019). Titanium dioxide nanoparticles induce apoptosis by interfering with EGFR signaling in human breast cancer cells. Environ. Res..

[15] Compaoré M., Lamien C.E., Lamien-Meda A., Vlase L., Kiendrebeogo M., Ionescu C., Nacoulma O. (2012). Antioxidant, xanthine oxidase and lipoxygenase inhibitory activities and phenolics of *Bauhinia rufescens* Lam. (Caesalpiniaceae). Nat. Prod. Res..

[16] Tucaliuc R.-A., Cotea V.V., Niculaua M., Tuchilus C., Mantu D., Mangalagiu I.I. (2013). New pyridazine–fluorine derivatives: Synthesis, chemistry and biological activity. Part II. Eur. J. Med. Chem..

[17] Ahmed A., Molvi K.I., Patel H.M., Ullah R., Bari A. (2020). Synthesis of novel 2, 3, 5-tri-substituted thiazoles with anti-inflammatory and antibacterial effect causing clinical pathogens. J. Infect. Public Health.

[18] Saniewski M., Horbowicz M., Kanlayanarat S. (2014). The Biological Activities of Troponoids and Their Use in Agriculture A Review. J. Hortic. Res..

[19] Cao F., Orth C., Donlin M.J., Adegboyega P., Meyers M., Murelli R.P., Elagawany M., Elgendy B., Tavis J.E. (2018). Synthesis and Evaluation of Troponoids as a New Class of Antibiotics. ACS Omega.

[20] Rout D., Dash U.C., Kanhar S., Swain S.K., Sahoo A.K. (2020). The modulatory role of prime identified compounds in the bioactive fraction of Homalium zeylanicum in high-fat diet fed-streptozotocin-induced type 2 diabetic rats. J. Ethnopharmacol..

[21] Bhowmik A., Das S., Sarkar W., Saidalavi K., Mishra A., Roy A., Deb I. (2021). Diastereoselective Spirocyclization via Intramo-lecular C (sp3)−H Bond Functionalization Triggered by Sequential [1,5]-Hydride Shift/Cyclization Process: Approach to Spiro-tetrahydroquinolines. Adv. Synth. Catal..

[22] Schoepfer J., Fretz H., Chaudhuri B., Muller L., Seeber E., Meijer L., Lozach O., Vangrevelinghe E., Furet P. (2002). Structure-based design and synthesis of 2-benzylidene-benzofuran-3-ones as flavopiridol mimics. J. Med. Chem..

[23] Jo G.-H., Choi I.-W., Jeong J.-W., Kim G.-Y., Kim J., Suh H., Ryu C.-H., Kim W.-J., Choi Y.H. (2014). Anti-inflammatory potential of newly synthesized 4-[(butylsulfinyl) methyl]-1, 2-benzenediol in lipopolysaccharide-stimulated BV2 microglia. Molecules.

[24] Clerici F., Gelmi M.L., Pellegrino S., Pocar D. (2009). ChemInform Abstract: Chemistry of Biologically Active Isothiazoles. ChemInform.

[25] Pein H., Ville A., Pace S., Temml V., Garscha U., Raasch M., Alsabil K., Viault G., Dinh C.-P., Guilet D. (2018). Endogenous metabolites of vitamin E limit inflammation by targeting 5-lipoxygenase. Nat. Commun..

[26] Quintanilla-Licea R., Vargas-Villarreal J., Verde-Star M.J., Rivas-Galindo V.M., Hernández D.T. (2020). Antiprotozoal Activity Against Entamoeba histolytica of Flavonoids Isolated from Lippia graveolens Kunth. Molecules.

[27] Zhang X., Blumenthal R.M., Cheng X. (2020). A Role for N6-Methyladenine in DNA Damage Repair. Trends Biochem. Sci..

[28] Buysse A.M., Yap M.C., Hunter R., Babcock J., Huang X. (2017). Synthesis and biological activity of pyridazine amides, hydrazones and hydrazides. Pest Manag. Sci..

[29] Mohammed A.E., Al-Qahtani A., Al-Mutairi A., Al-Shamri B., Aabed K. (2018). Antibacterial and cytotoxic potential of biosyn-thesized silver nanoparticles by some plant extracts. Nanomaterials.

[30] Hariharan D., Srinivasan K., Nehru L. (2017). Synthesis and characterization of TiO_2_ nanoparticles using Cynodon dactylon leaf extract for antibacterial and anticancer (A549 Cell Lines) Activity. J. Nanomed. Res..

[31] Manrique G.D., Lajolo F.M. (2002). FT-IR spectroscopy as a tool for measuring degree of methyl esterification in pectins isolated from ripening papaya fruit. Postharvest Biol. Technol..

[32] Santhoshkumar T., Rahuman A.A., Jayaseelan C., Rajakumar G., Marimuthu S., Kirthi A.V., Velayutham K., Thomas J., Venkatesan J., Kim S.-K. (2014). Green synthesis of titanium dioxide nanoparticles using Psidium guajava extract and its antibacterial and antioxidant properties. Asian Pac. J. Trop. Med..

[33] Hudlikar M., Joglekar S., Dhaygude M., Kodam K. (2012). Green synthesis of TiO_2_ nanoparticles by using aqueous extract of Jatropha curcas L. latex. Mater. Lett..

[34] Venkatachalam P., Sangeetha P., Geetha N., Sahi S.V. (2015). Phytofabrication of bioactive molecules encapsulated metallic silver nanoparticles from Cucumis sativus L. and its enhanced wound healing potential in rat model. J. Nanomed..

[35] Ravichandran S., Paluri V., Kumar G., Loganathan K., Venkata B.R.K. (2015). A novel approach for the biosynthesis of silver oxide nanoparticles using aqueous leaf extract ofCallistemon lanceolatus(Myrtaceae) and their therapeutic potential. J. Exp. Nanosci..

[36] Ramadan A.R., Yacoub N., Amin H., Ragai J. (2009). The effect of phosphate anions on surface and acidic properties of TiO_2_ hydrolyzed from titanium ethoxide. Colloids Surf. A Physicochem. Eng. Asp..

[37] Hair M., Tripp C. (1995). Alkylchlorosilane reactions at the silica surface. Colloids Surf. A Physicochem. Eng. Asp..

[38] Maurya A., Chauhan P., Mishra A., Pandey A.K. (2012). Surface functionalization of TiO_2_ with plant extracts and their combined antimicrobial activities against E. faecalis and *E. coli*. J. Res. Updates Polym..

[39] Sharma M., Behl K., Nigam S., Joshi M. (2018). TiO_2_-GO nanocomposite for photocatalysis and environmental applications: A green synthesis approach. Vacuum.

[40] Phromma S., Wutikhun T., Kasamechonchung P., Eksangsri T., Sapcharoenkun C. (2020). Effect of Calcination Temperature on Photocatalytic Activity of Synthesized TiO_2_ Nanoparticles via Wet Ball Milling Sol-Gel Method. Appl. Sci..

[41] Yan Y., Shi W., Peng W., Lin Y., Zhang C., Li L., Sun Y., Ju H., Zhu J., Ma W. (2019). Proton-free electron-trapping feature of titanium dioxide nanoparticles without the characteristic blue color. Commun. Chem..

[42] Humayun M., Raziq F., Khan A., Luo W. (2018). Modification strategies of TiO_2_ for potential applications in photocatalysis: A critical review. Green Chem. Lett. Rev..

[43] Hanaor D.A.H., Sorrell C.C. (2010). Review of the anatase to rutile phase transformation. J. Mater. Sci..

[44] Muhammad A., Sirat H.M. (2013). COX-2 inhibitors from stem bark of *Bauhinia rufescens* Lam. (Fabaceae). EXCLI J..

[45] Guo Y., Pei Y., Li K., Cui W., Zhang D. (2020). DNA N6-methyladenine modification in hypertension. Aging.

[46] Kanoujia J., Singh M., Singh P., Parashar P., Tripathi C.B., Arya M., Saraf S.A. (2016). Genipin crosslinked soy-whey based bio-active material for atorvastatin loaded nanoparticles: Preparation, characterization and in vivo antihyperlipidemic study. RSC Adv..

[47] Joyce P., Ulmefors H., Maghrebi S., Subramaniam S., Wignall A., Jõemetsa S., Höök F., Prestidge C.A. (2020). Enhancing the Cellular Uptake and Antibacterial Activity of Rifampicin through Encapsulation in Mesoporous Silica Nanoparticles. Nanomaterials.

[48] Gao L., Hu Y., Hu D., Li Y., Yang S., Dong X., Alharbi S.A., Liu H. (2020). Anti-obesity activity of gold nanoparticles synthesized from Salacia chinensis modulates the biochemical alterations in high-fat diet-induced obese rat model via AMPK signaling pathway. Arab. J. Chem..

[49] Rayalam S., Della-Fera M.A., Baile C.A. (2008). Phytochemicals and regulation of the adipocyte life cycle. J. Nutr. Biochem..

[50] Zhang G., Sun Q., Liu C. (2016). Influencing Factors of Thermogenic Adipose Tissue Activity. Front. Physiol..

[51] Brandão B.B., Poojari A., Rabiee A. (2021). Thermogenic Fat: Development, Physiological Function, and Therapeutic Potential. Int. J. Mol. Sci..

[52] Kang B., Kim C.Y., Hwang J., Jo K., Kim S., Suh H.J., Choi H.S. (2019). Punicalagin, a pomegranate-derived ellagitannin, sup-presses obesity and obesity-induced inflammatory responses via the Nrf2/Keap1 signaling pathway. Mol. Nutr. Food Res..

[53] Choi M., Mukherjee S., Yun J.W. (2021). Anthocyanin oligomers stimulate browning in 3T3-L1 white adipocytes via activation of the β3-adrenergic receptor and ERK signaling pathway. Phytotherapy Res..

[54] Nakamura K., Fuster J.J., Walsh K. (2013). Adipokines: A link between obesity and cardiovascular disease. J. Cardiol..

[55] Katiyar P., Yadu B., Korram J., Satnami M.L., Kumar M., Keshavkant S. (2020). Titanium nanoparticles attenuates arsenic toxicity by up-regulating expressions of defensive genes in *Vigna radiata* L.. J. Environ. Sci..

[56] Yagoub A.A., Alshammari G.M., Subash-Babu P., Mohammed M.A.A., Yahya M.A., Alhosain A.I. (2021). Synthesis of Ziziphus spina-christi (Jujube) Root Methanol Extract Loaded Functionalized Silver Nanoparticle (ZS-Ag-NPs); Physiochemical Characterization and Effect of ZS-Ag-NPs on Adipocyte Maturation, Adipokine, and Vascular Smooth Muscle Cell Interaction. Nanomaterials.

[57] McCoy M.K., Cookson M.R. (2011). DJ-1 regulation of mitochondrial function and autophagy through oxidative stress. Autophagy.

[58] Dinkova-Kostova A.T., Kostov R.V., Kazantsev A.G. (2018). The role of Nrf2 signaling in counteracting neurodegenerative diseases. FEBS J..

[59] Subash-Babu P., Al-Saran N., Alshammari G.M., Al-Harbi L.N., Alhussain M.H., Shamlan G., AlSedairy S.A., Alshatwi A.A. (2021). Evaluation of Biosafety, Antiobesity, and Endothelial Cells Proliferation Potential of Basil Seed Extract Loaded Organic Solid Lipid Nanoparticle. Front. Pharmacol..

[60] Yuan J.S., Reed A., Chen F., Stewart C.N. (2006). Statistical analysis of real-time PCR data. BMC Bioinform..

[61] Shi H., Magaye R., Castranova V., Zhao J. (2013). Titanium dioxide nanoparticles: A review of current toxicological data. Part. Fibre Toxicol..

[62] Sadrieh N., Wokovich A.M., Gopee N.V., Zheng J., Haines D., Parmiter D., Siitonen P.H., Cozart C.R., Patri A.K., McNeil S.E. (2010). Lack of significant dermal penetration of titanium dioxide from sunscreen formulations containing nano-and submicron-size TiO_2_ particles. Toxicol. Sci..

[63] Tsyganova N.A., Khairullin R.M., Terentyuk G.S., Khlebtsov B., Bogatyrev V.A., Dykman L.A., Erykov S.N., Khlebtsov N. (2014). Penetration of Pegylated Gold Nanoparticles Through Rat Placental Barrier. Bull. Exp. Biol. Med..

[64] Song B., Liu J., Feng X., Wei L., Shao L. (2015). A review on potential neurotoxicity of titanium dioxide nanoparticles. Nanoscale Res. Lett..

