# Supplementary figures and images for: Inhibition of Lipid Accumulation and Adipokine Levels in Maturing Adipocytes by Bauhinia rufescens (Lam.) Stem Bark Extract Loaded Titanium Oxide Nanoparticles
Source: Molecules. 2021 Nov 29;26(23):7238. doi: 10.3390/molecules26237238 (PMC8659042; doi:10.3390/molecules26237238)

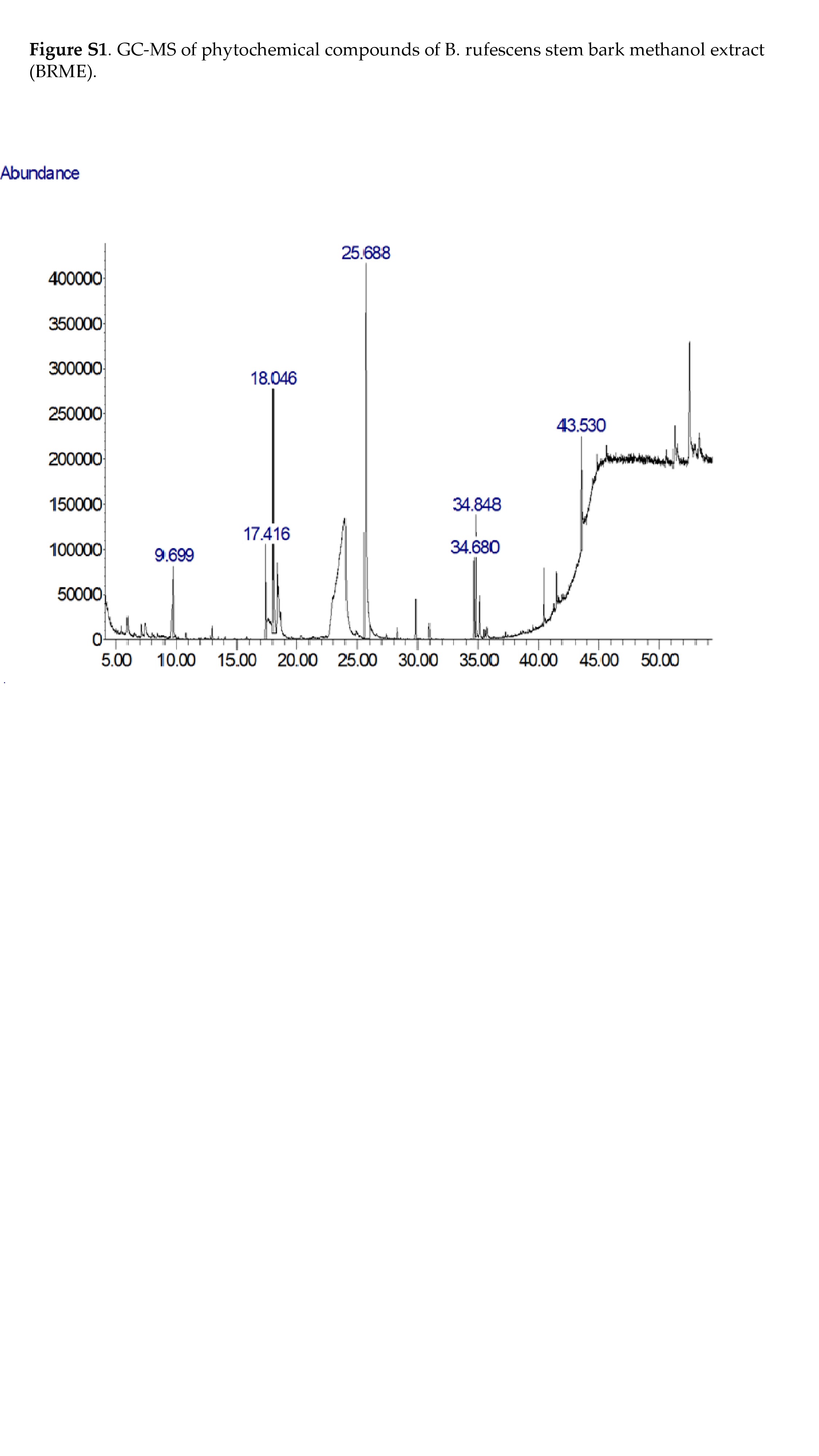

Supplement: Supplementary file 1 [file molecules-26-07238-s001.zip › p1fjjkrh9org2qmi17ui1k7qsph4-0.jpg]

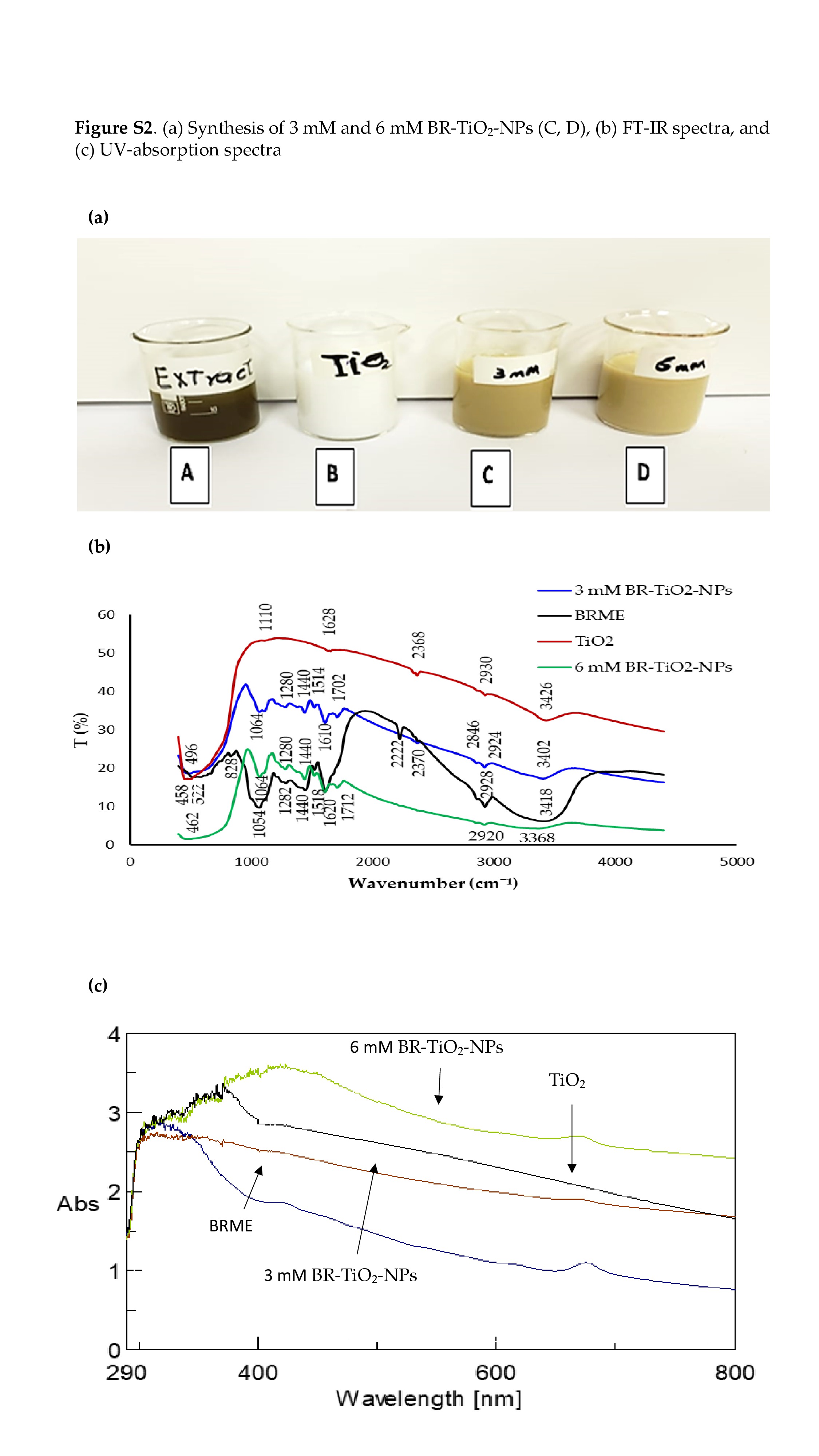

Supplement: Supplementary file 1 [file molecules-26-07238-s001.zip › p1fjjkrh9org2qmi17ui1k7qsph4-1.jpg]

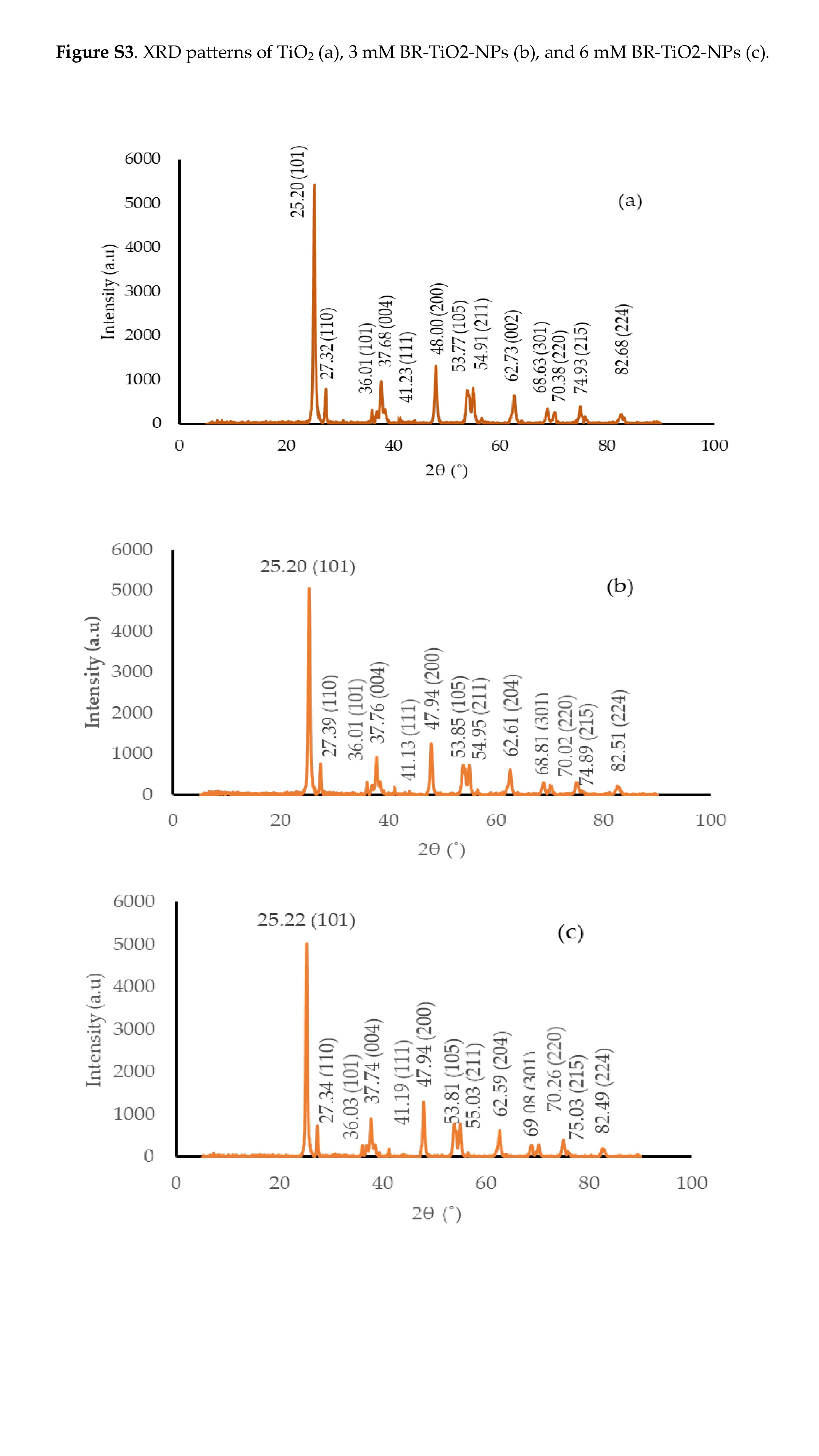

Supplement: Supplementary file 1 [file molecules-26-07238-s001.zip › p1fjjkrh9org2qmi17ui1k7qsph4-2.jpg]

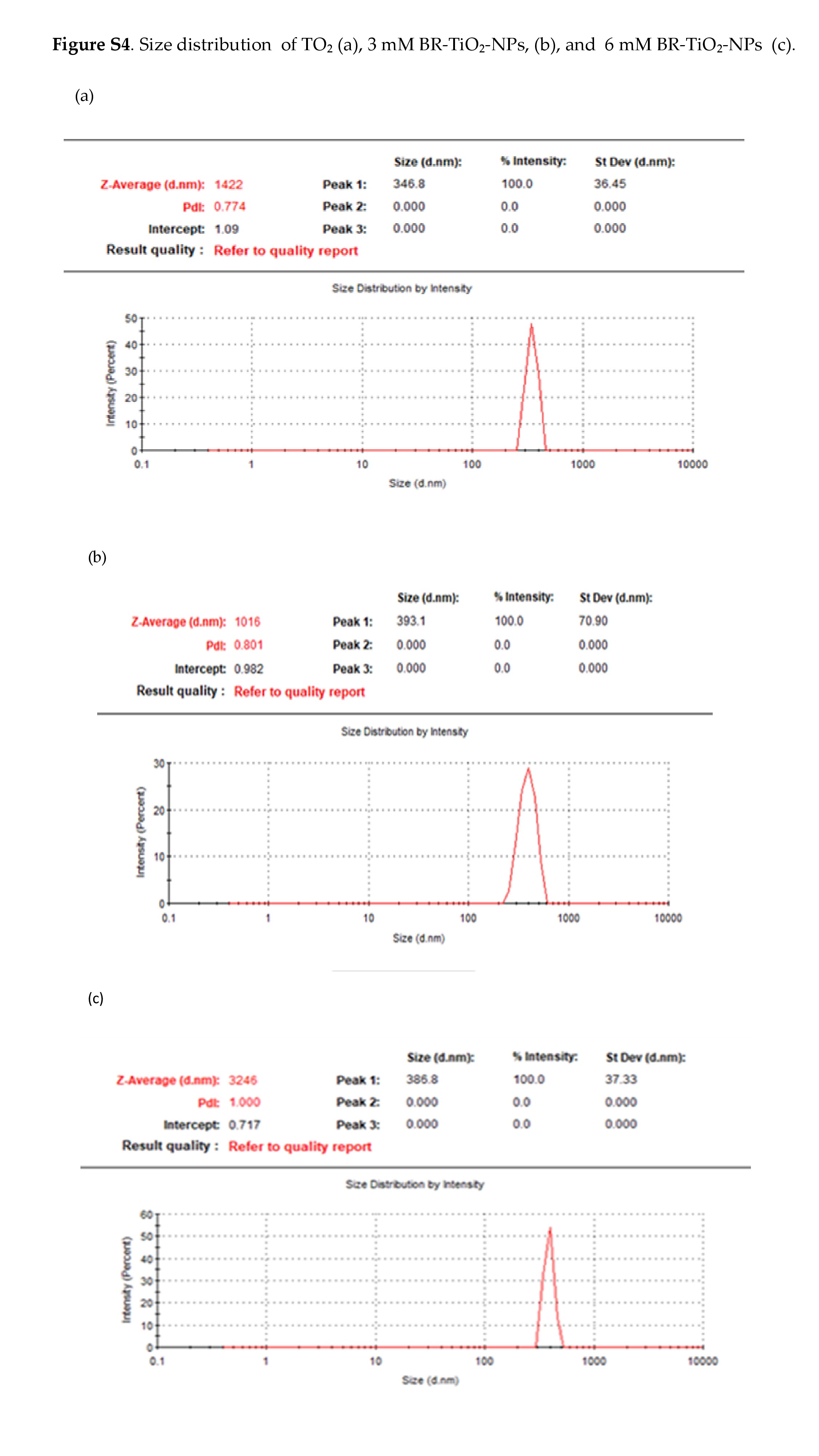

Supplement: Supplementary file 1 [file molecules-26-07238-s001.zip › p1fjjkrh9org2qmi17ui1k7qsph4-3.jpg]
